# *Persea americana* Glycolic Extract: *In Vitro* Study of Antimicrobial Activity against *Candida albicans* Biofilm and Cytotoxicity Evaluation

**DOI:** 10.1155/2015/531972

**Published:** 2015-10-29

**Authors:** D. Jesus, J. R. Oliveira, F. E. Oliveira, K. C. Higa, J. C. Junqueira, A. O. C. Jorge, G. N. Back-Brito, L. D. Oliveira

**Affiliations:** Laboratory of Microbiology and Immunology, Department of Biosciences and Oral Diagnosis, Institute of Science and Technology, Universidade Estadual Paulista (UNESP), 12245000 São José dos Campos, SP, Brazil

## Abstract

This study evaluated the antifungal activity of *Persea americana* extract on *Candida albicans* biofilm and its cytotoxicity in macrophage culture (RAW 264.7). To determine the minimum inhibitory concentration (MIC), microdilution in broth (CLSI M27-S4 protocol) was performed. Thereafter, the concentrations of 12.5, 25, 50, 100, and 200 mg/mL (*n* = 10) with 5 min exposure were analyzed on mature biofilm in microplate wells for 48 h. Saline was used as control (*n* = 10). After treatment, biofilm cells were scraped off and dilutions were plated on Sabouraud dextrose agar. After incubation (37°C/48 h), the values of colony forming units per milliliter (CFU/mL) were converted to log_10_ and analyzed (ANOVA and Tukey test, 5%). The cytotoxicity of the *P. americana* extract was evaluated on macrophages by MTT assay. The MIC of the extract was 6.25 mg/mL and with 12.5 mg/mL there was elimination of 100% of planktonic cultures. Regarding the biofilms, a significant reduction (*P* < 0.001) of the biofilm at concentrations of 50 (0.580 ± 0.209 log_10_), 100 (0.998 ± 0.508 log_10_), and 200 mg/mL (1.093 ± 0.462 log_10_) was observed. The concentrations of 200 and 100 mg/mL were cytotoxic for macrophages, while the concentrations of 50, 25, and 12.5 mg/mL showed viability higher than 55%.

## 1. Introduction

According to ANVISA [1], phytotherapics are medicines in which the active raw material is vegetables, such as fresh plant, plant drug, or its secondary products as extract, tincture, oil, and others such as juice and wax, obtained by adequate techniques. They are used for prophylactic, curative, palliative, or diagnosis purposes. The traditional phytotherapics medicines are synthesized from medicinal plants used traditionally by a population, with no risk to health, confirmed by toxicological studies, and demonstration of efficacy through ethnopharmacological surveys, or data obtained from the technoscientific documentation and indexed publications [1]. The use of these medicines is recognized by the World Health Organization (WHO); however, it is recommended that scientific studies be carried out to prove its effectiveness. Recent studies have demonstrated antimicrobial and anti-inflammatory efficacy of numerous plant extracts [2–4]. In 2013, Oliveira et al. [5] evaluated by microdilution method the antimicrobial activity of glycolic extracts of* Equisetum arvense* L.,* Glycyrrhiza glabra* L.,* Punica granatum* L., and* Stryphnodendron barbatimam* Mart. against* Staphylococcus aureus*,* Staphylococcus epidermidis*,* Streptococcus mutans*,* Candida albicans*,* Candida tropicalis,* and* Candida glabrata*, with all these extracts being effective against the microorganisms tested. Furthermore, they evaluated the cytotoxicity of plant extracts in RAW 264.7 mouse macrophages by MTT and colorimetric method for quantification of IL-1*β* and TNF-*α* cytokines. The study found that* G. glabra* extract exhibited the lowest cytotoxicity and* E. arvense* extract was the most cytotoxic. In 2014, Oliveira et al. [6] evaluated the antimicrobial activity of the extract of* Arctium lappa* L. against* S. aureus*,* S. epidermidis, Streptococcus mutans*,* C. albicans*,* C. tropicalis,* and* C. glabrata* in planktonic cultures and biofilm. The extract of* A. lappa* L. at the concentration of 250 mg/mL was microbicide for all tested microorganisms in the planktonic culture and significantly effective in reducing biofilms of these microorganisms.

Several plants have been the focus of studies to scientifically prove their many beneficial effects and promote better indication as an alternative therapy.* P. americana*, known as avocado tree, is an evergreen tree belonging to Lauraceae family, native of Central America, and currently distributed in tropical and subtropical regions. The plant drug of avocado tree consists of dried leaves containing at least 0.4% of total flavonoids expressed in apigenin and 0.14% of essential oil [7].

The fruit is widely exploited as food and its leaves are used in traditional medicine, fact found in ethnobotanical survey carried out by Rosas-Piñón et al. [8] in three distinct regions in Mexico, where the leaves are used in the treatment of oral infections and caries control. These authors analyzed the aqueous and alcoholic extracts of avocado leaf on* S. mutans* and* Porphyromonas gingivalis*, with satisfactory results only against* S. mutans* for both extracts. Lu et al. [9] evaluated the antimicrobial activity of methanolic extract of avocado green pulp against* Mycobacterium tuberculosis*, resulting in a minimum inhibitory concentration of 15 *μ*g/mL, which is considered very satisfactory as opposed to the resistance of this microorganism to different types of conventional antibiotics. In 2002, Adeyemi et al. [10] evaluated the anti-inflammatory and analgesic potential of the aqueous extract of avocado leaves in mice and found inhibition of pain and inflammation in a dose-dependent manner.

Guzmán-Rodríguez et al. [11] analyzed the antimicrobial potential of peptides derived from* P. americana* on* Escherichia coli*,* S. aureus,* and* C. albicans*. The results demonstrated antimicrobial effects for* E. coli* and* S. aureus*; however, activity on* C. albicans* was not detected. On the other hand, Leite et al. [12], analyzing the methanolic extract of* P. americana*, verified antimicrobial action in the planktonic culture strains of* Candida* spp.,* Cryptococcus neoformans,* and* Malassezia pachydermatis*.

Taking into consideration the resistance of microorganisms to antibiotics, it becomes necessary to study alternative methods such as plant extracts, and with the popular therapeutic use of avocado tree and some studies that suggest its antimicrobial and anti-inflammatory effects, it is of interest to study this extract in order to expand its therapeutic indication.

Many plant extracts have different therapeutic purposes, so that the studies with natural substances represent an important research field that may bring great benefits to dental therapy, being important to study their antimicrobial effects on dental-interest microorganisms and their possible cytotoxic effects. These studies are relevant to prove the various beneficial effects of these extracts, which can be further inserted in dental use formulations such as medications and intracanal irrigators, mouth rinses, toothpastes, and so on.

Thus, the objective of this study was evaluating the* in vitro* antifungal activity of* P. americana* extract on biofilm of* C. albicans* ATCC 18804 and its cytotoxicity in macrophage culture (RAW 264.7).

## 2. Materials and Methods

*P. americana* glycolic extract was provided by company Mapric (São Paulo, SP, Brazil) at the concentration of 200 mg/mL in propylene glycol.

In order to determine the minimum inhibitory concentration (MIC), broth microdilution method was used, according to CLSI [13], in accordance with norm M27-S4 protocol. Initially,* C. albicans* was cultured on Sabouraud dextrose (Himedia) for 24 h at 37°C. A standard solution containing 1 × 10^6^ cells/mL was prepared with spectrophotometer (Micronal B-582, São Paulo, SP, Brazil). Thereafter, this solution was diluted 1 : 50, followed by a 1 : 20 dilution to obtain a suspension of approximately 5 × 10^2^; to 2.5 × 10^3^; cells/mL.

Ten serial 1 : 2 dilutions were made from the extract into a 96-well plate (from 200 to 0.5 mg/mL) with 100 *μ*L of extract in 100 *μ*L of culture medium RPMI 1640 (Himedia) buffered with MOPS (Sigma Aldrich, St. Louis, Missouri, USA) at pH 7.0 ± 0.1. Subsequently, 100 *μ*L of the standardized suspension of the yeast was added to each well of 96-well plate. A well for positive control (medium with inoculum) and another well for negative control (medium alone) were included. The plates were incubated for 24 h at 37°C. The MIC was determined in the well of lowest concentration, in which turbidity was not observed. Aiming to find the minimum fungicidal concentration (MFC), 100 *μ*L of MIC, a concentration above and one below were seeded on Sabouraud dextrose plates and incubated at 37°C for 48 h.

### 2.1. Antimicrobial Activity in Biofilm

A standard strain of* C. albicans* (ATCC 18804) was used. The inoculum was standardized in sterile saline (NaCl 0.9%) containing 10^7^ cells/mL with spectrophotometer. Two hundred microliters of the inoculum was added in each well of a 96-well microplate. The microplate was incubated for 1.5 h (37°C under agitation of 75 rpm) for the initial adherence. Thus, the saline was taken, 200 *μ*L of YNB broth was added, and the microplate was incubated in the same conditions of initial adherence for 48 h. The medium was replaced every 24 h. After biofilm formation, the wells were divided into 6 experimental groups (*n* = 10), and from these 6 groups, 5 had contact with different concentrations of the extract, according to the results obtained in the planktonic assay: 12.5 mg/mL; 25 mg/mL; 50 mg/mL; 100 mg/mL; 200 mg/mL, during periods of 5 min. The control group was maintained in sterile saline. Posteriorly, these solutions were discarded, the biofilms were washed with sterile saline, and the samples were taken to the ultrasonic homogenizer (Sonopuls HD 2200, 50 W, Bandelin Electronic, Heinrichstraße, Berlin, Germany) for 30 s with approximately 25% of power to disaggregate the biofilm. There were four decimal dilutions of inoculum suspensions, which were seeded in duplicate (100 *μ*L of each dilution) in Sabouraud dextrose plates. These plates were incubated for 48 h at 37°C. Then, colony forming units per milliliter (CFU/mL) were counted and the results were converted to log_10_⁡ [14].

### 2.2. Cell Culture [5, 6]

Macrophages cell line RAW 264.7 was used, from the Rio de Janeiro Cell Bank, Associação Técnico Científica Paul Ehrlich (APABCAM, Rio de Janeiro, RJ, Brazil), cultured in Dulbecco modified Eagle medium (DMEM, LGC Biotechnology, Cotia, SP, Brazil), supplemented with 10% Fetal Bovine Serum (FBS, Invitrogen, Grand Island, NY, USA), and maintained under humidified atmosphere at 37°C and 5% CO_2_. An automatic cell counter (Countess, Invitrogen, Paisley, UK) was used to find the number of viable cells.

### 2.3. Cytotoxicity Testing (MTT Assay)

In 96-well microplates (TPP), 200 *μ*L of DMEM + 10% FBS containing 2 × 10^4^ viable cells per well was added. After incubation at 37°C, 5% CO_2_ for 24 hours for adhesion of the cells, the supernatant was discarded and* P. americana* extract diluted in DMEM + 10% FBS was added, in different concentrations, performing 11 experimental groups (*n* = 8): 0.2 mg/mL, 0.39 mg/mL, 0.78 mg/mL, 1.56 mg/mL, 3.13 mg/mL, 6.2 mg/mL, 12.5 mg/mL, 25 mg/mL, 50 mg/mL, 100 mg/mL, and 200 mg/mL. For growth control, culture medium with cell suspension was used and, for negative control, culture medium without cells was used. After 5 min of exposure, cell viability was measured by MTT assay. The supernatant was discarded, the wells were washed with sterile Phosphate Buffer Saline (PBS, Cultilab, Brazil), and it was added to the solution of MTT [3-(4,5-dimethylthiazol-2-yl)2,5-diphenyltetrazolium, Sigma Aldrich], at the concentration of 0.5 mg/mL. 12 repetitions for each group were performed. After incubation for 1 hour at 37°C, the solution was removed and 100 *μ*L of dimethyl sulfoxide (DMSO, Sigma Aldrich) was added. The plates returned to incubator for 10 minutes protected from light and were subsequently brought to the shaker for another 10 minutes. The absorbance of the wells was read under the wavelength of 570 nm in a microplate spectrophotometer (Bio-Tek, Winooski, Vermont, USA). The values of optical densities (OD) were converted into percentage of cell viability, compared with the control group [5].

### 2.4. Statistical Analysis

The data obtained were statistically analyzed by ANOVA and Tukey test, with significance level of 5% (*P* ≤ 0.05), through the statistical program GraphPad Prism 5.0.

## 3. Results

In planktonic cultures, the extract of avocado tree showed antimicrobial activity for* C. albicans* with minimum inhibitory concentration (MIC) at 6.25 mg/mL and minimum microbicidal concentration (MMC) at 12.5 mg/mL.

**Figure 1:**
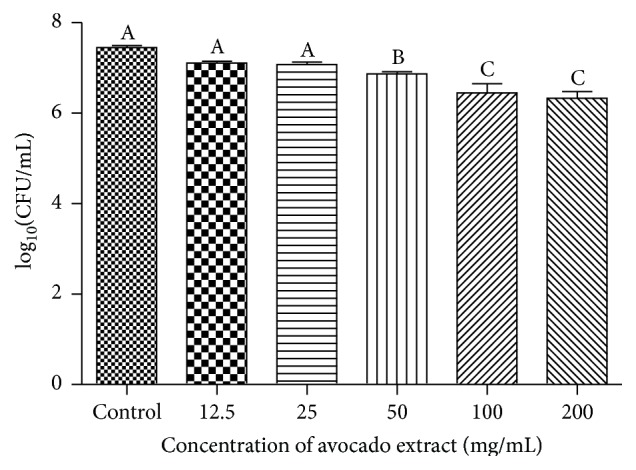
Number of CFU/mL (log_10_⁡) obtained in the untreated group (control 0.9% NaCl) and treated group with different concentrations of the extract for 5 min (*n* = 10). ANOVA and Tukey test (*P* < 0.05). Different letters indicate statistically significant differences.

*C. albicans* biofilms exposed for 5 min to different concentrations of the extract had significant reduction (*P* < 0.001) in the CFU/mL, regarding the control group, with the concentration of 50 mg/mL of* P. americana*, which reduced by 0.580 log_10_⁡ ± 0.209; in other words, this reduction corresponds to 74% (Figure 1).

**Figure 2:**
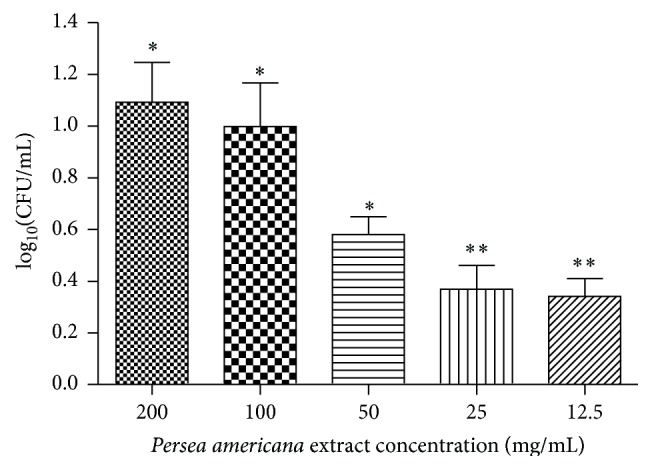
Reduction of* C. albicans* biofilm in the treated groups (*n* = 10). ^*∗*^Reduction statistically significant. ^*∗∗*^Reduction not statistically significant.

The greatest reduction in CFU/mL of* C. albicans* biofilm was obtained with the concentration of 200 mg/mL of the extract, with a reduction in the number of CFU/mL of 1.093 log_10_⁡ ± 0.462 compared to the positive control group biofilm, which had no contact with the extract (Figure 2). At the concentration of 100 mg/mL the reduction of* C. albicans* was 0.998 log_10_⁡ ± 0.508 CFU/mL, which was statistically similar to the value obtained at the concentration of 200 mg/mL (*P* > 0.05). At concentrations of 25 and 12.5 mg/mL, microbial reduction was not statistically different from the control group (*P* > 0.05).

**Figure 3:**
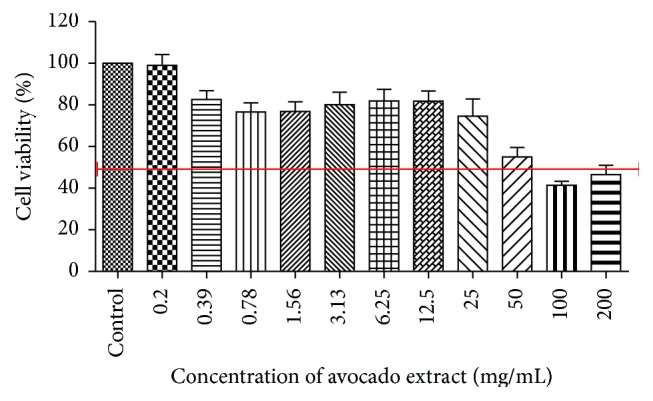
Cell viability of different concentrations of avocado comparing to control (*n* = 8).

Regarding the cytotoxicity test of* P. americana* extract, it can be observed that all concentrations of the extract, except 0.1 mg/mL, promoted reduction compared to control (*P* < 0.05), and the concentrations of 200 mg/mL and 100 mg/mL were the most cytotoxic ones for macrophages. The concentration of 50 mg/mL promoted cell viability around 55% and, from 25 mg/mL of the extract, cell viability was higher than 70%. The cell viability extract concentration *x* values are shown in Figure 3, where the red line marks the limit of cell viability 50%.

## 4. Discussion

The glycolic extract of avocado* P. americana* showed effective antifungal activity against* C. albicans* with MIC of 6.25 mg/mL and CMM of 12.50 mg/mL in planktonic cultures and caused a significant reduction of the biofilm from the concentration of 50 mg/mL, which could reduce by 0.580 log_10_⁡ ± 0.209, representing a reduction of 74%. These results agree with Leite et al. [12], showing that the methanol and hexane extracts of avocado seed had significant antifungal action in five* Candida* species and two species of* Cryptococcus neoformans* and* Malassezia pachydermatis*.

The potential antifungal activity of avocado extract is relevant, since in the last 30 years there has been a significant increase in fungal infections in humans due mainly to the increased incidence of immunosuppressive diseases such as HIV, therapies affecting the immune system, such as chemotherapy, and use of broad-spectrum antibiotics [15].

We highlight the antifungal action of avocado extract on* C. albicans* since most fungal infections that affect man have as etiological agent fungi of the genus* Candida* and most isolates of human candidiasis are from* C. albicans*. Studies show that* C. albicans* resistance to antifungal drugs is due to biofilm formation [16, 17] and more precisely to the presence of the matrix, which prevents the penetration of the drugs into the biofilm and limits their action of fungus cells [18]. This scenario shows the demand for antifungal agents that have effective action on biofilms of* C. albicans*, as found in this study with glycolic extract of* P. americana*.

Several studies extracted chemical constituents from various parts of avocado, finding compounds of the classes of alkanes [19, 20], alkaloids, flavonoids, and steroids, among others. From the leaves of avocado a compound called persin [21] was extracted, with a chemical structure similar to a fatty acid, which showed antifungal activity against* Colletotrichum gloeosporioides* [22]. Furan derivatives were also isolated from avocado plant and were shown to have antibacterial, antifungal, and insecticide action [23]. Rodríguez-Carpena et al. [24] analyzed the antibacterial action of methanol extracts of peel, pulp, and seed of avocado fruit over five strains of Gram-positive and Gram-negative bacteria, finding greater action on Gram-positive bacteria. On the other hand, Guzmán-Rodríguez et al. [11] found that peptides extracted from the avocado fruit (*P. americana*) showed antimicrobial activity on* Escherichia coli* and* S. aureus* but were not effective against* C. albicans*. Even though the same plant (*P. americana*), different extracts or extracted products have different antimicrobial results, what is a remarkable point to be considered to expand the studies with a focus on its therapeutic use.

Regarding the cytotoxic test, it can be seen that the extract higher concentrations (10% and 20%) caused significant cell death compared to control; however, at the concentration of 5% that in the antimicrobial tests showed a significant reduction in the* C. albicans *biofilm, the viability of RAW 264.7 macrophages in contact for 5 min was greater than 55%. As the main focus of the study is a possible therapeutic use for the extract in mouthwashes and toothpastes (external use), the concentration of 5% promoted good results on the analysis of antimicrobial activity and cell viability. On the other hand, when the focus indication is, for example, cancer treatment, this plant has shown interesting results in cytotoxicity. Avocado fruit is rich in phytochemicals, which play an important role in inhibition of growth of cancer cells [25]. Khalifa et al. [26] examined the effect of aqueous extracts from* P. americana* fruit and from leaves of* Tabernaemontana divaricata*,* Nerium oleander,* and* Annona cherimola* (positive control) in* Vicia faba* root cells. The roots of* Vicia faba* were soaked in plant extracts dilutions of 100, 1.250, 2.500, 5.000, 10.000, and 20.000 ppm for 4 and 24 h. All treatments resulted in a significant reduction in the mitotic index in a dose-dependent manner, and* P. americana* showed the highest cytotoxic effects with the increase of the concentration.* P. americana* treatment showed the highest cytotoxic effect on cancer cells, prophase cell percentage increased linearly with the applied concentration, and no micronuclei were detected. This study shows that root tip assay of beans can be used in initial screening for new plant extracts to validate their use as candidates for containing active cytotoxic agents against malignant cells. This will greatly help in exploring new plant extracts as drugs for cancer treatment.

Thus, it can be seen that the studies with* P. americana* are still at the beginning and that many biological actions of its constituents need to be analyzed in order to expand its therapeutic indication in several areas of health, including dentistry because, with their potential antifungal effect, this extract may in a near future be used as an alternative substance/adjuvant in the treatment of oral candidiasis.

## 5. Conclusion

Glycolic extract of* P. americana* showed significant antifungal activity against* C. albicans* biofilm and the concentration of 5% (50 mg/mL) presented the best results on the analysis of antimicrobial activity and cell viability.
